# Ritualized embodiment and moral self-cultivation: a Confucian model of psychological transformation

**DOI:** 10.3389/fpsyg.2026.1879160

**Published:** 2026-07-23

**Authors:** Yukun Mei, Sarawadee Phuchomsri, Jiyu Li

**Affiliations:** 1 School of Music and Dance, Xihua University, Chengdu, China; 2 Faculty of Fine and Applied Arts, Khon Kaen University, Khon Kaen, Thailand

**Keywords:** affect regulation, Confucian self-cultivation, culturally mediated self-regulation, embodied cognition, habit formation, moral psychology, philosophical psychology, theoretical psychology

## Abstract

This article develops a cross-cultural theoretical model of moral-psychological transformation by reinterpreting Confucian self-cultivation as a system of ritualized embodiment. Situated within theoretical and philosophical psychology, the article asks how bodily discipline, ritual form, and culturally mediated repetition may reorganize attention, affect, conduct, habit, and moral agency. Drawing on embodied cognition, moral psychology, ritual theory, cultural psychology, and Confucian philosophical discourse, the study proposes the Confucian Embodied Self-Cultivation Model. The model is grounded in close readings of selected classical formulations, especially keji fuli wei ren (克己复礼为仁) in the Analects and wu bu jing, yan ruo si (毋不敬,俨若思) in the Liji, and is historically illustrated through late-imperial lineage-based ancestral ritual practices, with Huizhou lineages used as a strategic analytic case rather than a representative sample of all Confucian traditions. The model identifies five interrelated dimensions: embodied moral agency, ritualized attention and affect regulation, repeated embodied self-cultivation, immanent psychological transformation, and cultural-institutional mediation. It argues that Confucian ritual should not be understood only as symbolic representation, ethical convention, or religious expression, but also as a culturally organized technology of self-regulation, habit formation, public accountability, and moral subject formation. By reframing Confucian cultivation in psychological terms, the article contributes to theoretical and philosophical psychology by expanding accounts of selfhood, moral agency, and psychological change beyond individualist, cognitivist, and belief-centered models. It concludes by formulating testable propositions for future research on embodied cognition, moral development, ritual participation, affect regulation, and culturally mediated self-cultivation.

## 1 Introduction

In recent decades, theoretical and philosophical psychology has increasingly challenged models of mind that treat cognition, agency, and moral judgment as primarily abstract, internal, and individual. Embodied cognition, cultural psychology, and practice-oriented accounts of selfhood have shown that psychological life is shaped through perception, posture, gesture, affect, repeated action, and socially organized forms of participation (Johnson, 1987; Lakoff and Johnson, 1999; Markus and Kitayama, 1991; Barsalou, 2008; Gallagher, 2005). From this perspective, the formation of a moral self cannot be reduced to belief, cognition, or decision-making alone; it also depends on trained bodily dispositions and culturally stabilized practices.

Despite this development, cross-cultural models of moral-psychological transformation remain insufficiently integrated into mainstream theoretical psychology. Much psychological theorizing continues to privilege individual self-regulation, reflective choice, internal motivation, or explicit judgment, while giving less attention to ritualized and institutionally mediated forms of self-formation. The problem is not simply that culture is underrepresented as content, but that cultural practice is often treated as background context rather than as a mechanism through which attention, affect, habit, and agency are organized. Confucian self-cultivation offers a useful case for addressing this gap because it conceptualizes the self as relational, embodied, practice-dependent, and normatively formed through ritual propriety (li 礼), reverential attentiveness (jing 敬), and cultivated humanity (ren 仁).

Confucian traditions have often been classified either as ethical philosophy or as a contested form of non-theistic religion. This article does not attempt to settle that classificatory debate. Instead, it asks what Confucian self-cultivation can contribute to theoretical and philosophical psychology. The central claim is that Confucian cultivation provides a model of psychological transformation in which attention, affect, habit, and bodily conduct are reorganized through ritualized practice and social mediation. Religious and cosmological language remains relevant because Confucian cultivation is oriented toward a normatively charged moral order, but the present article translates that orientation into psychological terms: embodied transformation, culturally scaffolded self-regulation, and practice-based moral agency.

A related terminological issue concerns the meaning of jiao (教) and jiaohua (教化) in Confucian contexts. These terms may be rendered as “teaching,” “moral education,” “civilizing transformation,” or, in some debates, as part of the question of whether Confucianism should be understood as a religious tradition. The present article does not resolve this semantic and classificatory issue. Instead, it treats jiao/jiaohua as a vocabulary of formative practice: a culturally organized process through which persons are shaped by ritual norms, embodied discipline, textual learning, and social participation. This interpretation is compatible with the psychology-oriented aim of the article, because the central question is not whether Confucianism is best classified as religion or moral education, but how Confucian practices of formation may reorganize attention, affect, habit, and moral agency.

This article therefore proposes the Confucian Embodied Self-Cultivation Model. The model conceptualizes Confucian cultivation as a five-layer process: embodied moral agency, ritualized attention and affect regulation, repeated embodied self-cultivation, immanent psychological transformation, and cultural-institutional mediation. It does not present Confucianism as a homogeneous or transhistorical tradition. Rather, it abstracts a historically situated mechanism through which ritualized embodiment may generate durable moral-psychological dispositions.

The analysis combines two evidentiary registers. First, it offers close readings of selected Confucian classical passages, especially Analects 12.1 and Liji, Qu Li I.1, to reconstruct the textual grammar of bodily restraint, ritual reintegration, reverential attention, and cultivated moral agency. Second, it examines lineage-based ancestral ritual practices in late-imperial local society, especially Huizhou lineages, as a historical illustration of how ritual norms became enforceable, repeatable, and embodied. Huizhou is not treated as statistically representative of Confucianism; it is used as a strategic analytic case that makes the proposed mechanism unusually visible through dense records of lineage organization, ritual rules, sanctions, ancestral halls, and corporate resources.

The research questions are accordingly psychological and theoretical: How does Confucian ritual construct the body as a medium of moral agency rather than as a passive biological substrate? How do ritualized rules of seeing, hearing, speaking, moving, dressing, arriving, bowing, and participating function as mechanisms of attention regulation, affective discipline, and habit formation? How can a tradition that does not center a personal supernatural agent produce psychological transformation through embodied practice? And how can such a model contribute to comparative theories of embodied cognition, moral development, and culturally mediated self-regulation?

Methodologically, the article is a theoretical and interpretive study rather than an experimental report. It is best understood as a hypothesis-and-theory contribution: it constructs a conceptual model from philosophical texts and historically documented practices, then formulates implications that can be examined through future comparative, observational, developmental, educational, or experimental research. The historical materials are not used to generalize statistically, but to clarify how psychological mechanisms may become socially organized, externally scaffolded, and institutionally durable.

The article contributes to theoretical and philosophical psychology in three ways. First, it expands accounts of self-regulation by showing how disciplined bodies and ritual scripts can function as externalized regulators of attention, affect, and conduct. Second, it contributes to moral psychology by conceptualizing virtue not as an isolated internal trait, but as an embodied and socially recognizable capacity stabilized through practice. Third, it enriches cross-cultural psychology by offering a non-Western model of selfhood and transformation in which cultural institutions and ritualized participation are not peripheral contexts but constitutive conditions of psychological formation.

The argument proceeds as follows. Section 2 situates the study within embodied cognition, ritual theory, moral psychology, cultural psychology, and culturally mediated self-regulation. Section 3 explains the textual and historical materials, including the analytic use of Huizhou lineage ritual. Section 4 formulates the Confucian Embodied Self-Cultivation Model. Section 5 reconstructs its operational logic through close reading and institutional illustration. Section 6 discusses its implications, limits, and testable propositions for psychological theory. Section 7 concludes by clarifying the model's contribution to comparative accounts of psychological transformation.

## 2 Embodied cognition, ritual practice, and psychological transformation

The present study is grounded in the convergence between embodied cognition, philosophical psychology, moral psychology, and ritual theory. Embodied approaches reject the idea that cognition is only a computational operation detached from bodily life. Instead, cognition is grounded in sensorimotor capacities, bodily schemas, affective orientation, and situated interaction (Varela et al., 1991; Johnson, 1987; Lakoff and Johnson, 1999; Wilson, 2002; Barsalou, 2008; Gallagher, 2005). This orientation is especially relevant for traditions of self-cultivation, where psychological transformation is not only described in propositional terms but enacted through posture, gesture, attention, speech, and repeated forms of conduct.

For moral psychology, the key issue is how moral dispositions become stable. Models of self-regulation emphasize monitoring, feedback, inhibition, goal-directed adjustment, and the stabilization of behavior over time (Carver and Scheier, 1982; Bandura, 1991; Haidt, 2001). Yet such models are often framed at the level of the individual organism or the reflective agent. Confucian ritual practice suggests a complementary possibility: self-regulation may be distributed across ritual forms, bodily scripts, hierarchical relations, spatial arrangements, calendars, public correction, and institutional memory. In this sense, ritual is not merely a cultural expression of psychology; it can be theorized as one of the mechanisms through which psychological capacities are formed, monitored, and made socially recognizable.

Ritual theory helps clarify this mechanism. Ritualized practice does more than represent values. It organizes attention, regulates affect, marks distinctions, establishes authority, and produces durable dispositions through repetition and correction (Bell, 1992, pp. 90–91; Bourdieu, 1977, p. 72). When ritual prescribes where the body stands, how it bows, when it speaks, what it wears, and how it responds to others, it functions as a technology of embodied regulation. Such regulation is psychological because it shapes attention, emotion, impulse control, social perception, and moral agency.

This argument can be further clarified by psychological anthropology and the anthropology of ritual. Bourdieu's concept of habitus is useful because it explains how socially structured practices can become durable bodily dispositions rather than remaining explicit rules or conscious intentions. Csordas's work on embodiment and “somatic modes of attention” is especially relevant to the present model, because it describes culturally elaborated ways of attending to and with one's body in an intersubjective world (Csordas, 1990, 1993). From this perspective, Confucian ritual does not merely transmit beliefs or symbolize values; it trains how bodies attend, restrain, respond, and become available to moral correction. Ritualized seeing, hearing, speaking, moving, bowing, dressing, arriving, and occupying ranked positions may therefore be interpreted as culturally organized forms of somatic attention. This anthropological perspective reinforces the psychological claim advanced here: ritualized embodiment functions as a mediating mechanism through which attention, affect, habit, and moral agency are formed within socially organized practice.

This framework also makes it possible to reconsider forms of transformation that do not center private belief or supernatural intervention. Confucian cultivation is oriented toward a moral-cosmic vocabulary, but its operative psychological mechanism lies in disciplined participation, ritualized attention, affect regulation, and embodied moral formation. The relevant question for theoretical psychology is therefore not whether Confucianism fits a theistic definition of religion, but how Confucian practice organizes the transformation of persons through bodily, social, and institutional means.

Confucian studies has long emphasized the relational and embodied character of personhood. Philosophical interpretations of Confucian selfhood stress that the person is formed through roles, relationships, ritual conduct, and cultivated responsiveness rather than through autonomous interiority alone (Hall and Ames, 1995, pp. 13–20; Ames, 1998). East Asian body-mind theories similarly treat cultivation as a disciplined transformation of bodily and psychological capacities (Yuasa, 1993, pp. 3–18). These insights remain underdeveloped within psychological theory, where Confucian materials are often treated as cultural background rather than as a source of conceptual innovation.

The gap addressed here is therefore not simply the marginal position of Confucianism within theoretical and philosophical psychology, but the limited integration of Confucian self-cultivation into theories of psychological change. Existing models of self-regulation often explain moral transformation through individual monitoring, inhibition, motivation, or reflective judgment. They give less systematic attention to how self-regulation may be scaffolded by ritual forms, bodily scripts, social correction, architectural space, and institutional repetition. This article synthesizes embodied cognition, ritual theory, cultural psychology, and Confucian thought to construct a model in which bodily discipline, ritualized attention, affect regulation, habit formation, and institutional mediation operate together. The model is not offered as a universal description of all Confucian traditions, but as an analytic framework for understanding how ritualized embodiment can organize moral-psychological change.

The proposed model can also be situated in relation to several existing psychological frameworks. Like self-regulation theory, it concerns monitoring, inhibition, feedback, and behavioral adjustment, but it shifts the emphasis from individual internal control to culturally distributed regulation through ritual scripts, public correction, spatial arrangement, and institutional repetition (Carver and Scheier, 1982). Like theories of habit formation, it stresses repetition and stabilization over time, but it adds that ritual repetition is normatively charged, socially visible, and institutionally supported (Wood and Neal, 2007). In relation to predictive processing, ritual may be understood as providing patterned expectations for perception and action, reducing uncertainty in morally significant social situations by specifying how bodies should see, hear, speak, move, dress, bow, and respond (Clark, 2013). Situated cognition and ecological psychology are also relevant, because Confucian ritual formation occurs not in an isolated mind but within structured environments: ancestral halls, ritual calendars, ranked positions, genealogical documents, and sanction regimes provide affordances for attention, affect regulation, and moral conduct (Gibson, 1979; Hutchins, 1995). The Confucian Embodied Self-Cultivation Model therefore does not replace these approaches; rather, it extends them by showing how self-regulation, habit formation, and moral agency can be culturally organized through ritualized embodiment and institutional mediation.

## 3 Confucian texts and lineage ritual as historical-psychological materials

Textual note: Citations of the Lunyu 论语, Liji 礼记, and Zhongyong 中庸 follow widely used Zhonghua Shuju editions, specifically Yang (1980) for the Analects and Ruan Yuan (ed.), Shisanjing Zhushu (1980 [1815]) for the Liji tradition. Passages are cited using widely adopted chapter/section conventions to ensure verifiability (e.g., Analects 12.1; Liji, Qu Li I.1; Zhongyong 20). For the Daxue 大学 chapter within the Liji, citations refer to the chapter text without assuming a unified subsection numbering across editions. English renderings are my own unless otherwise noted. For philological control, key passages were checked against widely used annotated editions and established translations, including Yang Bojun's commentary, Legge's missionary-era rendering (Legge, 1893), Slingerland's modern scholarly translation with annotation (Slingerland, 2003), and Ames and Rosemont's philosophical translation (Ames and Rosemont, 1998). These translations are used interpretively to support the article's theoretical argument rather than to resolve philological disputes. Classical passages cited by text title and section number are therefore linked to the editions listed in the reference list under Yang (1980) and Ruan (1980 [1815]). Core Confucian terms are provided in pinyin with Chinese characters at first occurrence—xiushen 修身, li 礼, ren 仁, jing 敬, dao 道, and shengren 圣人—and subsequently appear in pinyin or English for readability. References to lineage practices employ standard historical terminology (e.g., zongzu 宗族, zupu 族谱, citang 祠堂, sitian 祀田, and fayin 罚银).

Before proceeding to the analysis of lineage-based ancestral practice, a methodological clarification is necessary. This article does not treat Huizhou lineages as statistically representative of “Confucianism” across all regions and periods. Rather, Huizhou functions as a strategic case and an analytic anchor for making the proposed mechanism of embodied self-cultivation empirically legible (Flyvbjerg, 2006; Yin, 2014, pp. 40–42). For a psychology-oriented theoretical article, the Huizhou materials are used to illustrate how ritualized bodies, repeated participation, public correction, and institutional enforcement can stabilize attention, affect, and moral disposition over time.

Late-imperial Huizhou offers three advantages for this purpose. First, its institutional density—corporate lineages, ancestral halls, ritual calendars, and office-based responsibilities (e.g., sinian 司年, menzhang 门长)—makes it possible to observe how ritual norms are translated into administrable obligations (attendance, precedence, timed presence, and sanctions). Second, its documentary richness—genealogies (zupu 族谱), lineage regulations, household codes, and archival devices, such as the “public box” (gongxia 公匣)—renders ritual continuity traceable and enforceable across generations. Third, its economic substrate—sacrificial lands (sitian 祀田) and earmarked ritual funds—underwrites calendrical repetition and helps insulate practice from fluctuations in individual piety. Together, these features allow the ritual-institutional configuration examined here to be observed at unusually high resolution.

Therefore, the argument presented in this article is a historically delimited analysis of a configuration in which embodied self-cultivation becomes observable, not a claim of national representativeness. At the same time, this study recognizes regional and historical variation in lineage organization and ritual practice. For example, in parts of North China, weaker lineage density and corporate strength often made intensive lineage-centered discipline less sustainable (Chang, 2010, p. 103). In coastal southeastern China (e.g., southern Fujian, Chaoshan), the entanglement of lineage ritual with temple networks and popular devotion may reconfigure how embodied discipline is produced and authorized. These differences do not undermine the present analysis; rather, they delineate its scope and identify promising directions for comparative research.

If embodiment-centered approaches foreground practice as analytically significant, Confucian ritual traditions provide a particularly illuminating field in which such theoretical insights become historically observable. Among the diverse ritual repertoires associated with Confucian culture, lineage-based ancestral organization zongzu (宗族) constitutes one of the most stable and institutionally embedded domains through which bodily discipline, moral cultivation, attention regulation, and social authority converge. While ritual carries symbolic dimensions, ancestral practice also reveals how normative order is enacted through repetitive bodily techniques, spatial arrangements, and institutional continuity. This pattern becomes particularly visible in the local society of southeastern China, where Huizhou lineage documentation provides unusually dense institutional records.

In the present study, li (礼) is not employed as a singular or purely ceremonial term, but as an analytical continuum comprising three interrelated dimensions. First, li functions as a normative discipline, articulated in classical discourse as a prescriptive grammar governing comportment, hierarchy, and moral restraint. Second, li appears as an institutionalized ritual repertoire, encompassing concrete ceremonial forms—most centrally here, lineage-based ancestral rites—embedded in spatial, calendrical, and genealogical infrastructures. Third, li extends into everyday bodily technique, shaping habitual gestures, postural regulation, affective restraint, and the rhythmic patterning of speech and interaction. These dimensions do not operate independently; rather, they form a continuous process through which normative prescription becomes institutional script and is subsequently internalized as embodied disposition. In this sense, li operates simultaneously as ethical grammar, ritual structure, and corporeal habitus.

Ancestral ritual in late-imperial China was not a form of occasional commemoration but a structured regime of conduct embedded in lineage institutions. Manuals and regulations preserved in genealogies (zupu 族谱), together with the spatial organization of ancestral halls (citang 祠堂), prescribed kneeling sequences, hierarchical positioning, and movement protocols. Such acts were not only expressions of reverence but disciplinary techniques through which participants learned to inhabit hierarchical order and regulate affect. Importantly, these prescriptions were enforceable rather than hortatory; attendance, timing, and comportment were treated as regulated competencies rather than optional sentiments. He (2010) situates these mechanisms within Huizhou local governance, showing how corporate institutions translated ritual norms into administrable rules, while McDermott (2020, pp. 60–124) demonstrates that the ancestral hall functioned as an organizational node linking ritual order with finance, credit, and collective management—rendering governance routines and ritual practice mutually constitutive.

The persistence of this regime relied on an economic substrate that underwrote repetition. Huizhou regulations earmarked income from sacrificial lands (sitian 祀田) for annual rites:

“一祀田所入,充每年祭祀之费,岁不可缺。”

Author's translation: “Income from sacrificial lands is used to cover the annual expenses of sacrifice; it must not be lacking in any year.”

Source: IAHS, Fudan University (2012), Ming–Qing Huizhou de cun gui min yue yu xiangcun zhili ziliao xuanbian «明清徽州的村规民约与乡村治理资料选编», entry “Qing Yongzheng Xiuning Mingzhou Wu shi zongzu jia gui / 清雍正休宁茗洲吴氏宗族家规,” p. 40.

Here, ritual appears not as episodic devotion but as a funded institution in which calendrical recurrence, material provisioning, and ranked participation reinforced one another. Equally crucial was documentary infrastructure. Beyond genealogies and hall rules, Huizhou lineages maintained archival devices, such as the “public box” (gongxia 公匣), which stored contracts and regulations as lineage property (He, 2010, p. 42). These mechanisms rendered ritual continuity traceable and enforceable; written documentation was not external to embodiment but a condition of its reproducibility.

Within such settings, bodily comportment was specified in operational terms: kneeling at designated moments, bowing according to rank, and aligning bodies with ancestral tablets and senior members. The convergence of space (citang), inscription (zupu), office-based responsibility (e.g., sinian 公匣, menzhang 门长), and sanction (fayin 罚银) suggests that culturally authorized legitimacy emerged through institutional continuity and repetition, without depending solely on doctrinal assertion. Through recurrent participation, individuals enacted a disciplined self, structured by filial piety (xiao 孝), hierarchical awareness, and corporeal restraint.

Although this discussion foregrounds lineage ritual, it does not isolate the zongzu sphere from state structures. Late-imperial regimes operated within a state-local assemblage: lineage institutions were locally administered yet remained legible within state ritual vocabularies. Therefore, the analysis presented in this article addresses the meso-level, where canonical norms became enforceable obligations—attendance, precedence, calendrical cycles, and sanctions—mediated by corporate organizations. Local elites acted as brokers who mobilized Confucian language to legitimate discipline while implementing it through lineage mechanisms. Lineage authority thus functioned neither as a replica of state ritual nor as an autonomous domain, but as an embedded extension of a broader ritual-moral order reproduced through embodied participation.

This configuration should not be taken to represent Confucianism in its entirety; temple rites, state sacrifices, educational institutions, and modern moral education constituted additional domains. Yet lineage practice offers a particularly clear lens because it locates psychological formation within everyday organization rather than elite ceremony alone. Regional variability further cautions against homogenization; scholarship notes differences in lineage density and hall organization across North China, Jiangnan, and South China (Chang, 2010, p. 103).

From a psychological perspective, ancestral ritual illustrates how the body can become both a site and an instrument of culturally mediated moral regulation. Its significance is not reducible to abstract metaphysical belief; rather, it becomes socially effective through disciplined repetition, regulated affect, spatialized hierarchy, and public accountability. Spatial arrangement directs attention; ritual sequence scripts bodily conduct; sanctions supply external feedback; calendrical repetition stabilizes habit; and genealogical documentation preserves normative expectations across generations. Huizhou lineage ritual is therefore not used here merely as historical background, but as an example of how cultural institutions can organize the formation of attention, affect, conduct, and moral agency over time. Authority is articulated discursively and consolidated performatively; transformation is realized through qualitative change within participation. The ritualized body becomes the locus where moral cultivation, social order, and culturally mediated authority intersect, providing an empirical anchor for interpreting Confucian self-cultivation as a practice-centered system of psychological formation grounded in historically situated institutions.

## 4 The Confucian embodied self-cultivation model

The preceding discussion indicates that Confucian self-cultivation cannot be adequately interpreted as abstract moral doctrine alone. What becomes analytically visible is a patterned process in which bodily discipline, ritual regulation, and institutional continuity may jointly organize durable changes in attention, affect, conduct, habit, and moral agency. The Confucian Embodied Self-Cultivation Model proposed here describes this process as a theoretical model of psychological transformation rather than as a classificatory verdict about Confucianism as religion.

At its most fundamental level, the model begins from embodied moral agency. In Confucian traditions, the body is not merely a biological substrate or an external instrument of an already formed moral consciousness. It is the medium through which moral attention, restraint, reverence, and responsiveness become trainable. Classical discourses of xiushen (修身) and li (礼) articulate cultivation as a process enacted through posture, comportment, speech, timing, and regulated conduct. Corporeality is therefore not the exterior of moral psychology but one of its operative sites.

The second dimension is ritualized attention and affect regulation. Ritual functions as a formative and performative technology: it scripts conduct, coordinates bodies, regulates emotional expression, and makes behavior publicly legible and correctable. Representation and formation operate together. The analytical significance of ritual lies not only in what it symbolizes, but also in what it produces: stabilized attentional habits, regulated affect, relational orientation, and durable patterns of moral subjectivity.

The third dimension is embodied self-cultivation. Confucian cultivation does not rely primarily on private introspection or doctrinal assent. It becomes effective through repeated training at the level of sensory conduct, speech, movement, posture, deference, and relational timing. In psychological terms, this is a culturally organized form of self-regulation, habit formation, and moral learning. Moral agency is gradually produced as the person becomes capable of inhabiting ritual forms reliably and responsively.

The fourth dimension is immanent psychological transformation. Confucian transformation does not require escape from the world or dependence on a personal transcendent deity. Rather, it can be theorized as a qualitative shift within embodied existence: a transformation of disposition, perception, conduct, and ethical orientation. Ideals such as shengren 圣人 (sagehood) can therefore be interpreted as achieved states of cultivated psychological and moral orientation within practiced life.

The fifth dimension is cultural-institutional mediation. Ritual practice becomes durable when it is supported by social infrastructures such as lineage organizations, ancestral halls, ritual calendars, genealogical regulations, and economic resources. These institutions do not merely provide the context for psychological formation; they organize repetition, feedback, accountability, and transgenerational continuity. They function as extended regulatory environments through which embodied self-cultivation becomes socially durable.

The model is not intended as a transhistorical essence of Confucianism. It is an analytical abstraction derived from a specific historical configuration: the intersection of classical textual discourse and lineage-based ritual institutions in late-imperial local society. It is most useful where ritual norms are repeatedly enacted, socially monitored, and institutionally supported. Other domains—state rites, temple networks, educational institutions, or modern ethical education—may assemble similar mechanisms differently and require separate analysis.

Viewed in this light, the model shifts the question from “Is Confucianism a religion?” to a more psychologically productive question: how may ritualized embodiment contribute to psychological transformation? The answer proposed here is that Confucian cultivation operates through a layered mechanism in which bodily regulation scripts attention and affect, repeated ritual participation stabilizes habits, and institutional mediation makes moral-psychological transformation durable and publicly recognizable.

## 5 The operational logic of Confucian embodied self-cultivation

**Figure 1:**
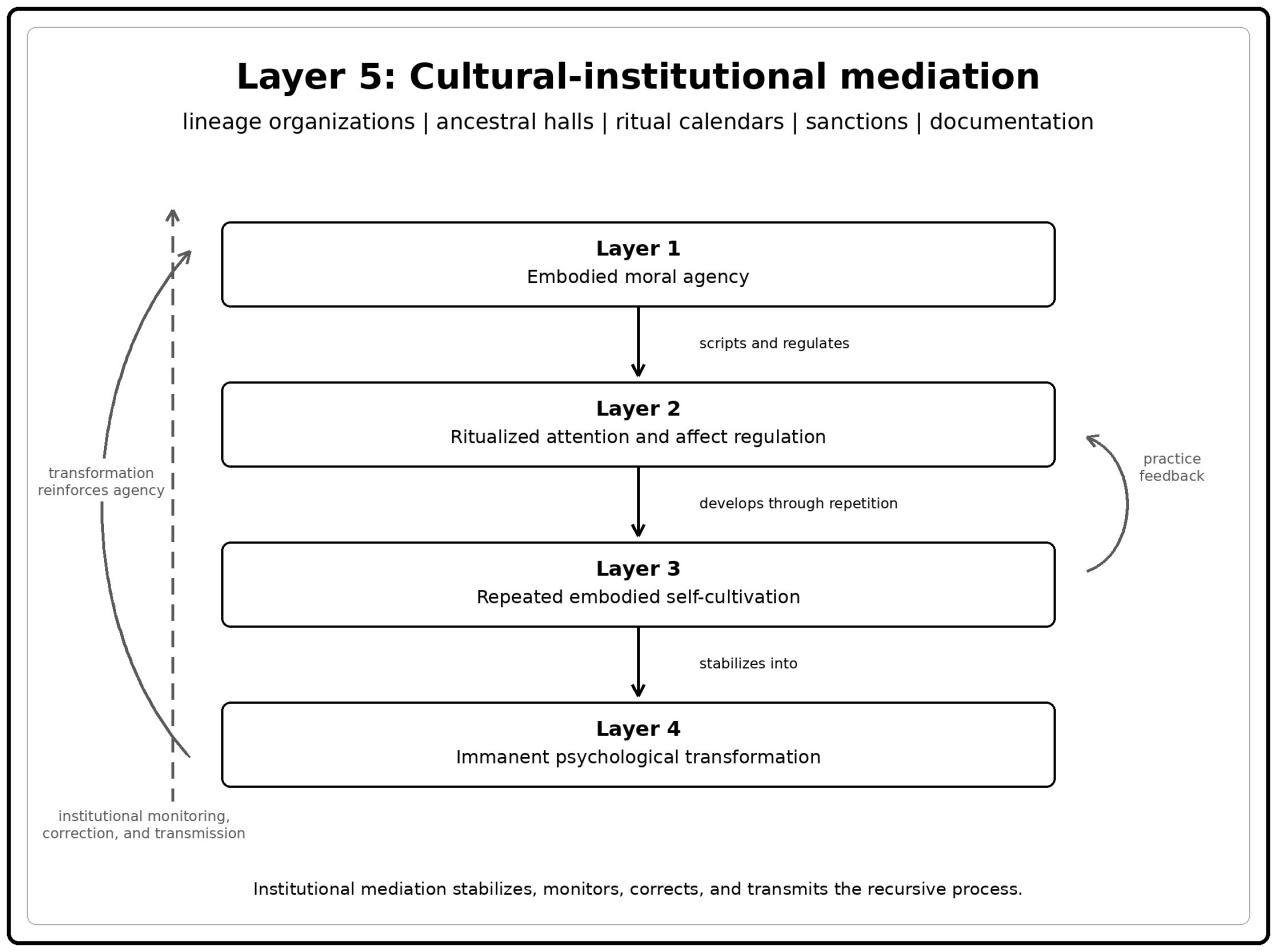
Confucian embodied self-cultivation model. The model is presented as a recursive rather than strictly linear process. Layer 5, cultural-institutional mediation, encompasses and interacts with the four inner layers through ritual calendars, spatial arrangements, documentary infrastructures, sanction regimes, and intergenerational participation. Embodied moral agency becomes trainable through ritualized attention and affect regulation; repeated embodied practice stabilizes these attentional and affective dispositions, and immanent psychological transformation, once formed, reinforces embodied moral agency. The feedback loops indicate that ritualized self-cultivation is sustained through continuous interaction among bodily discipline, affective-attentional regulation, repeated practice, psychological transformation, and institutional mediation.

Building on the preceding discussions, this section moves beyond isolated textual and institutional observations to examine how the diverse elements of Confucian practice—bodily discipline, ritual regulation, attention, affect, habit, and institutional continuity—cohere into an integrated psychological logic. Rather than introducing a pre-formed schematic model in advance, the analysis traces how these elements interrelate in practice, allowing the underlying structure to emerge from the conjunction of classical discourse and lineage-based ritual regimes. Figure 1 offers a schematic orientation to the model developed in this section; the five-part structure is summarized at the end of the section in (Table 1).

**Table 1:** Structural dimensions of the Confucian embodied self-cultivation model.

Layer	Core construct	Textual/historical anchor	Psychological function
Layer 1 Embodied moral agency	Body as moral-psychological medium	Zhongyong 20; Analects 12.1	Grounds moral agency in trained bodily conduct.
Layer 2 Ritualized attention and affect regulation	Ritual scripts perception, speech, movement, and demeanor	Liji, Qu Li I.1; Analects 12.1	Regulates attention, affect, conduct, and relational responsiveness.
Layer 3 Repeated embodied self-cultivation	Practice-based habit formation	Analects 1.1; Liji, Daxue	Stabilizes moral self-regulation through repetition and correction.
Layer 4 Immanent psychological transformation	Transformation of disposition, perception, and ethical orientation	Zhongyong 1	Frames transformation within practiced life rather than as abstract belief alone.
Layer 5 Cultural-institutional mediation	Extended regulatory environment	Huizhou lineage regulations, ancestral halls, calendars, sanctions, sitian 祀田, zupu 族谱, and gongxia 公匣	Provides feedback, accountability, recurrence, and transgenerational transmission.

This Table summarizes the analytic structure distilled from Sections 5.1 to 5.3. It presents five dimensions alongside their conceptual cores, key textual or historical anchors, and psychological functions. Classical Confucian passages are cited by text title and section number; the editions used are listed in the references under (Yang 1980) and Ruan (1980 [1815]). Historical institutional evidence is included where the argument concerns mediation, repetition, and enforcement. The table is intended as a comparative instrument: it renders the reconstructed operational logic legible across cases without implying a transhistorical essence.

The analytic structure distilled from Sections 5.1 to 5.3 is summarized in Table 1 at the end of Section 5.

### 5.1 Sustained close reading of the classical grounding

A particularly revealing formulation appears in Analects 12.1, where Confucius answers the question of ren (仁), not by offering a propositional definition but by giving a concise sequence of cultivation: keji fuli wei ren (克己复礼为仁). Read in context, the line is not a slogan but a compact articulation of how ren is to be made present through disciplined conduct by overcoming self-regarding inclinations (ji 己), returning to li, and thereby bringing ren about. This framing prepares the close reading that follows, in which the regulation of seeing, hearing, speaking, and moving (“非礼勿视/听/言/动”) is treated as the concrete pathway through which the text renders moral formation publicly legible and repeatable.

First, ke (克) names an active act of “overcoming” or “subduing,” not a mild tempering. In Analects 12.1, this verb signals that ren is not presented as a spontaneous inner state but as something achieved through deliberate regulation. The object of this overcoming is ji (己), which is here understood as a self-regarding inclination rather than a metaphysical self. In the immediate Analects context, ji is best taken not as an abstract metaphysical “self,” nor as a later Song-Ming reading that often reads the language of “desire” (yu 欲) into the term, but as self-interest, convenience, reactive emotions, and other tendencies that readily issue in unregulated conduct. The text itself specifies where such tendencies are to be met and checked: “Not in accordance with li, do not look; not in accordance with li, do not listen; not in accordance with li, do not speak; not in accordance with li, do not move” ((非礼勿视、非礼勿听、非礼勿言、非礼勿动). This fourfold regulation of seeing, hearing, speaking, and moving can be read in dialogue with the Daxue's macro-formulation of xiushen (修身), specifying self-cultivation at the level of everyday conduct. This reading follows the Analects' immediate framing of keji regarding regulated seeing, hearing, speaking, and moving, and does not presuppose later Song-Ming theories of yu (欲) or inward moral metaphysics. Read in this way, keji is articulated as a trainable capacity of restraint at the very points where conduct becomes visible—seeing, hearing, speaking, and moving—so that what is to be overcome is encountered in patterned comportment rather than in a purely inward psychological register.

Second, fu (复) marks a movement of “returning” or “restoring.” It does not simply mean “to follow” in the sense of external compliance, but indicates re-entry into an already recognized order of norms and forms. Here, li (礼) cannot be reduced to “good manners.” Within the present study, li names an integrated field that includes (i) a normative grammar of restraint and hierarchy, (ii) an institutional repertoire of rites, and (iii) embodied techniques through which such norms are learned and stabilized. To “return to li” (fuli) is thus to re-situate the body within a publicly legible and repeatable form of relational order—one in which posture, speech, timing, precedence, and deference are rendered correctable and accountable. This is why the Analects formulation is not merely negative (“overcome what is of oneself”) but re-integrative: restraint receives determinate direction through re-alignment with li as a shared form that organizes conduct within patterned relations (elder/junior, ruler/minister, teacher/student, and ancestor/descendant).

Third, the phrase wei ren (为仁) is grammatically decisive. Wei indicates making or bringing about; ren is presented as an achieved state rather than a private moral substance presumed to be present in advance. The sequence—keji → fuli → wei ren—thus conveys a formative logic: regulated conduct becomes ethically efficacious as it is gathered into ritual form, and ren names the outcome of this patterned re-formation. In this sense, the passage offers not a doctrinal definition of ren but a concise program of cultivation, which is expressed through a rule of conduct and its repetition. Moral authority here is generated through disciplined regulation of embodied comportment and re-inscription into li as a normative-ritual order, without being limited to cognitive assent to doctrinal propositions.

The wider sentence context reinforces the processual and socially expansive meaning of ren. Confucius adds the following: “If for 1 day a person can overcome the self and return to li, all under Heaven will ‘return' to ren” (一日克己复礼,天下归仁焉). In its immediate force, tianxia gui ren (天下归仁) indicates that ren is not confined to private inwardness; it becomes publicly manifest and socially effective through shared forms of conduct. The verb gui (归) is instructive here: it suggests a movement of turning-toward or coming-to—an orientation that others can recognize and to which they can respond—rather than naming a merely private moral feeling. Because the return is articulated through li, the transformation is rendered recognizable and comparable: it recalibrates expectations within concrete relations and provides a common measure for evaluating comportment in the surrounding community. Confucius then insists, “To be ren depends on oneself—how could it depend on others?” (为仁由己,而由人乎哉). This is not a claim of atomistic individualism but a demand for agency within a relational order: one becomes capable of ren by taking responsibility for disciplined conduct and for re-entry into the normative-ritual form of li.

Read in this way, keji fuli wei ren functions as a concise grammar for the formation of a cultivated person within a normatively structured configuration. It describes how a transformed agent may be produced through repeated regulation of embodied conduct and reintegration into li as a shared form, without treating transformation as a matter of private belief alone.

A complementary formulation appears in Liji, Qu Li I.1: wu bu jing, yan ruo si (毋不敬,俨若思). If Analects 12.1 foregrounds the sequence of restraint and return, this line clarifies the sustained disposition that makes such a sequence practicable over time. The construction wu bu (毋不) is rhetorically maximal: it does not recommend reverence in special moments, but demands that no act be without jing (敬). Jing here should not be treated as a vague inner sentiment. In ritual discourse, it names a cultivated stance of reverent attentiveness—an ethically charged vigilance that stabilizes posture, regulates affect, and keeps action continuously answerable to form. Therefore, Jing is the affective-attentional infrastructure of keji: without sustained reverential watchfulness, “overcoming the self” collapses into episodic effort or moral enthusiasm. With jing, restraint becomes a durable capacity.

The phrase yan (俨) then renders this reverent attentiveness publicly manifest as visible, repeatable comportment. Yan suggests a composed, dignified, and controlled bodily presence—an external seriousness that is not theatrical display but disciplined form. The final ruo si (若思) is equally important: “as if in thought.” It does not instruct constant abstract reasoning; rather, it prescribes a bodily demeanor that resembles concentrated reflection. In other words, the text binds “inner” orientation to “outer” form by requiring that the body present itself as if guided by reflective seriousness. This alignment is precisely what an embodied account of ritual requires: moral or normatively charged states are not accessed by introspection alone but are cultivated through publicly accountable bodily technique. Jing is made stable through yan; attentiveness is sedimented as posture; and reverence becomes a bodily style that can be repeated, recognized, and corrected.

Read together, Analects 12.1 and Liji, Qu Li I.1 form a coherent chain that supports the model's embodiment claim. Keji names the negative moment of discipline (interrupting impulse at the level of sensory and motor conduct). Jing names the positive, sustaining disposition (continuous reverential attentiveness that keeps the body aligned). Fuli names the reintegrative moment (returning bodily life to a ritual form that structures relations and hierarchy). Wei ren names the emergent outcome (ren as a constituted moral-psychological subjectivity rather than an untrained inner essence). In this integrated reading, “克己” is best understood as bodily restraint, “复礼” as ritual re-integration, and “仁” as the produced form of a cultivated self whose embodied life becomes consonant with a normative moral order.

Taken together, these formulations already sketch a trajectory of transformation that the model will later theorize as immanent psychological transformation, but they do so through the mundane mechanics of disciplined embodiment, attention regulation, and ritual form.

This is also where the linkage to xiushen (修身) becomes conceptually precise. The Daxue formulation that “from the Son of Heaven down to the common people, all must take self-cultivation as the foundation” (自天子以至于庶人,壹是皆以修身为本) can be read as the macro-frame, while keji/fuli/jing/yan specify the micro-mechanism through which cultivation operates in practice. Xiushen is not an abstract interior project; it is accomplished through repeated bodily regulation, ritual re-inscription, and the formation of a stable reverential disposition. In this sense, the classical texts do not merely endorse morality; they provide an operational grammar for how a cultivated person is formed through embodied discipline and ritualized reintegration.

The close reading of Analects 12.1 and Liji, Qu Li I.1 brings into view a patterned logic. Bodily restraint (keji), when reintegrated into ritual form (fuli) and sustained by reverential attentiveness (jing), yields an achieved moral orientation named ren. What is at stake here is not an abstract moral psychology detached from practice, but an operational sequence—discipline, ritualization, and transformation—articulated in and through regulated conduct. The question that follows is whether this sequence remains only a textual ideal, or whether it can be observed as a historically durable formation in the institutionalized ritual practices of local society.

### 5.2 Historical illustration: Huizhou lineage ritual regimes

Building on Sections 3 and 5.1, this section examines a single regulatory cluster—attendance rules and their sanction regime—as a concrete demonstration of how enforceable norms become bodily scripts and how repeated participation may sediment as jing (敬), a durable habitus of reverential attentiveness (Bourdieu, 1977, p. 72). The point is not to reduce ritual to coercion, but to show how social accountability, repetition, and bodily scripting can be theorized as mechanisms of psychological formation.

Huizhou corporate normative documents preserved in genealogical and associational compilations state attendance not as moral advice but as an enforceable duty. One lineage household rule requires household heads to lead their juniors to the ritual or meeting site in proper attire and within a fixed time window:

“各户长率子弟衣冠齐诣会所,限于辰时毕至。非病患、事故、远出,毋得偷怠因 循不至。”

Author's translation: “Each household head shall lead his sons and grandsons, properly dressed, to attend the meeting place; all must arrive by the chen hour. Unless due to illness, accident, or travel, no one may slack off or fail to attend.”

Source: IAHS, Fudan University (2012), Ming–Qing Huizhou de cun gui min yue yu xiangcun zhili ziliao xuanbian«明清徽州的村规民约与乡村治理资料选编», entry “Ming Longqing 6 Qimen Wentang xiangyue jiafa (Wentang Chen lineage) / 明隆庆六年祁门文堂 乡约家法（文堂陈氏宗族）”, p. 21.

A related association rule makes the collective requirement even more explicit and immediately attaches sanctions to absence:

“十二股齐到。如有不到者,公罚米六升交众,毋许入席……若有不到者,毋得散 胙。”

Author's translation: “All twelve branches must be present. If any are absent, they shall be publicly fined six sheng of rice and shall not be allowed to join the banquet; if anyone is absent, they may not receive the distributed sacrificial portions.”

Source: IAHS, Fudan University (2012), Ming-Qing Huizhou de cun gui min yue yu xiangcun zhili ziliao xuanbian«明清徽州的村规民约与乡村治理资料选编», entry “Daoguang 10 Jixi Taizi shen hui huigui / 清道光十年绩溪太子神会会规”, p. 32.

What matters here is the granularity with which these clauses regulate bodies. Yiguan qi yi (衣冠齐诣) does not merely recommend respect; it demands a visible bodily state—proper dress and ordered appearance—and a coordinated movement of bodies to a designated site. Xian yu chen shi bi zhi (限于辰时毕至) adds temporal discipline: punctuality is not optional but a timed obligation. The lineage hierarchy of mobilization is also built into the grammar of the rule (household heads leading sons and grandsons), turning intergenerational order into a repeatable circulation of bodies. In this sense, the regulations exemplify li as a ritual technology: they specify how the body must appear, where it must go, and when it must arrive, thereby producing disciplined participation through scripted conduct rather than exhortation to belief.

The sanction regime then secures this bodily scripting by operating on two registers at once. On the material register, the absentee is publicly fined a quantified commodity (gong fa mi liu sheng, 公罚米六升交众). The measurability of the penalty is analytically significant: it converts ritual absence into an administrable infraction and makes discipline reproducible across cases. On the social register, absence triggers exclusion from commensality (wu xu ru xi, 毋许入席) and from the distribution of sacrificial portions (wu de san zuo, 毋得散胙). Both sanctions target the body directly: they withdraw food, seating, and participation in the shared sensory-affective scene through which lineage solidarity and moral authority are enacted. Because the fine is public (gong, 公) and the exclusions are visible, punishment also functions as a reputational technology: it makes non-compliance legible to others and therefore harder to normalize. The operation of these rules makes the internal linkage analytically explicit: ritual scripting—through procedural norms of dress, movement, and timed presence—is stabilized by corporate mediation (fines, exclusions, and reputational sanctions), and, under calendrical recurrence, can sediment as durable dispositions of reverent attentiveness (jing), thereby linking institutional enforcement to embodied self-cultivation.

The analytic point is not that Huizhou lineages relied only on coercion, but that sanctioned obligations may stabilize repetition, and repetition is what makes internalization psychologically plausible. Under calendrical recurrence, institutionally required attendance may repeatedly train the body into a predictable regime of punctuality, solemn demeanor, and hierarchical responsiveness. Over time, what begins as external compliance may sediment as a dispositional orientation: continuous attentiveness and self-watchfulness, which the Liji names jing (敬). This claim is not an empirical generalization about all participants; it is a theoretical account of how institutionalized repetition can scaffold habit formation.

Therefore, the Huizhou attendance-sanction cluster does not merely document an isolated historical practice. It renders visible the operational logic distilled in the preceding close reading (Analects 12.1; Liji, Qu Li I.1): ritual form scripts embodied conduct, institutional enforcement stabilizes repetition, and repetition supports internalization. In this sense, the case supports an operational account of culturally mediated psychological transformation: moral-psychological change becomes intelligible as a shift from externally monitored compliance to embodied attentiveness, affect regulation, and habitual responsiveness.

Methodological note: The Confucian Embodied Self-Cultivation Model is advanced as an analytic construction rather than an empirical generalization. It is abstracted through conceptual synthesis across two evidentiary registers: (i) canonical formulations that ground the model's psychological functions at the level of textual discourse, and (ii) late-imperial lineage-based ritual regimes (especially Huizhou) where those functions become institutionally enforceable and historically durable. Accordingly, the model is not proposed as a transhistorical essence of Confucian tradition, nor does it exhaust the diversity of Confucian practices.

The model specifies a historically situated configuration in which embodied moral agency, ritualized attention and affect regulation, transformative practice, immanent psychological change, and cultural-institutional mediation cohere into a practice-based system of self-cultivation, summarized below in Table 1. On this basis, the next section distills the analytic structure that has emerged across both registers and articulates it in a concise schematic form.

### 5.3 Articulating the operational structure

The preceding close reading and historical illustration allow a more systematic articulation of the operational logic that has emerged across the analysis. What becomes visible is not a single doctrinal proposition but a configuration in which bodily discipline, ritual form, attention regulation, affective discipline, habit formation, and institutional continuity mutually reinforce one another. To clarify this configuration without treating it as a transhistorical essence, the following synthesis distills five analytic dimensions that recur across the two evidentiary registers.

At the level of embodied moral agency, the body is treated not as a merely biological substrate or a neutral instrument subordinated to moral consciousness, but as the operative medium through which Confucian cultivation becomes psychologically effective. Classical discourses of xiushen (修身) and li (礼) articulate cultivation as enacted through posture, comportment, and regulated conduct. In this sense, moral agency becomes observable through embodied formation grounded in corporeal discipline and ritual mediation.

At the level of mechanism, li functions as a formative technology that renders embodied conduct publicly legible, repeatable, and correctable. It operates across normative discipline, institutionalized ritual repertoires, and everyday bodily techniques shaped by ritual prescriptions. The point is not primarily what ritual symbolizes, but what it accomplishes psychologically: ritual scripts attention, affect, movement, speech, and relational timing through form, thereby producing structured subjectivity within shared—and, in some settings, enforceable—modes of participation.

At the level of practice, self-cultivation operates as embodied discipline. The mechanism becomes effective over time through repeated training and correction at the level of sensory conduct, speech, bodily movement, posture, and relational timing. Transformation is thus not reducible to doctrinal assent; it is realized as a reproducible capacity for regulated conduct cultivated through sustained practice.

At the level of transformation, Confucian cultivation is experienced as immanent psychological transformation. The shift occurs within embodied existence—disposition, comportment, perception, and ethical orientation—rather than through metaphysical escape. Ideals such as shengren (圣人) are approached as achieved states of cultivated orientation within practiced life, rather than as otherworldly endpoints.

At the level of system, the foregoing processes are stabilized by cultural-institutional mediation. Lineage organizations, ancestral halls, ritual calendars, documentary infrastructures, economic resources, and sanction regimes do not merely host practice; they operationalize repetition, authority, accountability, and transgenerational continuity, thereby making embodied formation durable and transmissible. In this configuration, transformation is constituted and maintained through disciplined corporeality, ritual regulation, public feedback, and institutional continuity rather than through private cognition alone.

As an analytical tool, the model is not proposed as a universal essence of Confucianism. Rather, it is distilled from historically situated configurations in which these analytic dimensions become structurally visible—most prominently, the conjunction of classical textual discourse and lineage-based ritual regimes. Its purpose is conceptual clarification: to specify how a culturally organized system of embodied practice can form persons by regulating attention, affect, conduct, and moral agency over time.

Table 1 summarizes these five analytic dimensions—embodied agency, mechanism, practice, transformation, and system—as a concise schematic of the operational structure distilled in this chapter. The table is not offered as a universal template or a definitional verdict about Confucianism, but as an analytic abstraction derived from the specific historical configuration examined here, and as a comparative instrument for tracing how analogous mechanisms are assembled—or disrupted—across different cultural and institutional ecologies.

## 6 Discussion: contributions to theoretical and philosophical psychology

Building on the textual reconstruction and historical illustration in Section 5, this discussion clarifies the theoretical implications of the Confucian Embodied Self-Cultivation Model. The model contributes to theoretical and philosophical psychology by specifying how psychological transformation can be theorized through embodied practice, ritualized attention, affect regulation, habit formation, and cultural-institutional mediation.

First, the model expands embodied cognition by showing that embodiment is not only a condition of perception and meaning, but also a mechanism of moral transformation. In the Confucian case, posture, gesture, timing, restraint, reverential demeanor, and repeated ritual participation are not peripheral expressions of internal states; they are techniques through which attention and affect are trained. This extends embodied approaches from cognition broadly conceived to the formation of moral agency.

Second, the model contributes to moral psychology by reframing virtue as a practice-stabilized capacity rather than an isolated internal trait. The close reading of Analects 12.1 and Liji, Qu Li I.1 suggests that moral agency emerges through a sequence of bodily restraint, ritual reintegration, reverential attention, and repeated practice. Ren, in this interpretation, is not simply a moral concept to be endorsed, but an achieved orientation made durable through embodied discipline.

Third, the model enriches theories of self-regulation. Psychological accounts of self-regulation often emphasize feedback, monitoring, inhibition, and goal adjustment. Confucian ritual practice suggests how these processes can be culturally distributed. Ritual rules, ancestral halls, calendars, household roles, public sanction regimes, and shared expectations externalize regulatory demands and make them repeatable. The self is therefore not formed only by internal control; it is also shaped by environments that script, monitor, and correct bodily conduct.

Fourth, the model offers an account of transformation that does not depend on theistic premises. In many psychological and religious frameworks, transformation is explained either through internal psychological change or through relation to a supernatural source. The Confucian configuration analyzed here suggests another pathway: immanent transformation through disciplined participation in a normatively charged moral order. Such transformation is psychological because it reorganizes disposition, attention, affect, and conduct; it is philosophical because it presupposes a particular account of personhood, agency, and moral order.

The model also generates testable propositions for future research. Proposition 1: ritualized bodily repetition may increase attentional stability toward normatively salient cues by linking perception, posture, and social expectation. Proposition 2: public and spatially organized ritual participation may strengthen the association between bodily posture, affective restraint, and perceived moral obligation. Proposition 3: institutionalized repetition, especially when supported by calendars, sanctions, and shared roles, may stabilize moral conduct more durably than isolated moral instruction alone. Proposition 4: traditions that embed moral ideals in bodily scripts may produce forms of self-regulation that differ from models based primarily on private belief, individual choice, or verbal teaching. Proposition 5: externalized correction and social accountability may accelerate the internalization of attentional and affective discipline. These propositions can be examined through cross-cultural comparison, observational studies of ritual practice, developmental studies of moral learning, educational interventions, or experimental simulations of embodied ritual cues. At the same time, the claims of the model must be bounded carefully.

Several limitations should be noted. First, the model is culturally specific. Although it identifies mechanisms that may be compared with other traditions of embodied self-cultivation, it is grounded in Confucian ritual discourse and should not be generalized as a universal model of moral psychology. Second, the historical illustration depends on the institutional density of late-imperial lineage society. Huizhou ancestral halls, lineage regulations, ritual calendars, sanction regimes, sacrificial lands, and documentary infrastructures made embodied repetition unusually visible, but other Confucian contexts may have assembled these mechanisms differently. Third, the model poses challenges for empirical operationalization. Classical terms such as li, jing, and ren cannot be translated directly into single psychological variables; they require multidimensional indicators involving attention, affect, comportment, relational responsiveness, perceived accountability, and moral agency. Fourth, ritual participation is difficult to reproduce in experimental settings because its psychological force depends on repetition, public visibility, spatial arrangement, social hierarchy, and institutional legitimacy. Future research should therefore combine observational, longitudinal, comparative, and experimental approaches rather than relying on laboratory designs alone.

Future empirical research may operationalize the model through several measurable domains. Ritualized attention could be examined through gaze orientation, attentional stability, responsiveness to ritual cues, or task-based measures of sustained attention in normatively structured settings. Affect regulation could be measured through self-reported emotional restraint, perceived reverence, physiological arousal, and behavioral indicators of composure. Embodied conduct could be assessed through posture, timing, bowing accuracy, movement coordination, and adherence to ritual sequence. Ritual participation could be operationalized through frequency, duration, role, perceived seriousness, and perceived public accountability. Habit formation could be assessed through consistency of conduct across repeated settings, automaticity of ritual response, and stability of embodied comportment over time. Moral agency could be examined through norm-sensitive judgment, responsibility attribution, prosocial decision-making, and willingness to regulate self-interest in relational contexts. These indicators would not exhaust the classical meanings of li, jing, or ren, but they would make the model empirically tractable for future psychological research. The possible operational indicators for future empirical research are summarized in Table 2.

**Table 2:** Possible operational indicators for future empirical research.

Model construct	Possible operational indicators
Ritualized attention	Gaze control, attentional stability, response to ritual cues, sustained attention in normatively structured settings
Affect regulation	Emotional restraint, self-reported reverence, physiological arousal, behavioral composure
Embodied conduct	Posture, timing, bowing accuracy, movement coordination, adherence to ritual sequence
Ritual participation	Frequency, duration, role, perceived seriousness, participation history
Public accountability	Perceived monitoring, correction, sanction awareness, reputational concern
Habit formation	Consistency of conduct across repeated settings, automaticity of ritual response, stability of embodied comportment
Moral agency	Norm-sensitive judgment, responsibility attribution, prosocial decision-making, regulation of self-interest

Future research can extend the framework in several directions. Comparative analysis across state rites, temple networks, educational institutions, and contemporary moral education can test how the same embodied mechanism changes when authority, venue, and enforcement differ. Developmental and educational psychology could examine how ritualized conduct shapes children's or students' moral attention and affective regulation. Cultural psychology could compare Confucian self-cultivation with Buddhist, Daoist, Christian, Islamic, or secular practices of discipline. Empirical work could also test whether embodied ritual cues affect attention, emotional restraint, social perception, or moral decision-making differently from verbal moral instruction. Such studies would transform the present model from a theoretical reconstruction into a broader research program on embodied moral-psychological transformation.

The Confucian case should therefore be understood not only as an illustration of general theories of embodied cognition and ritual formation, but also as a culturally specific permutation of those theories. It shows that the ritual formation of the self may operate through historically particular vocabularies, institutions, and bodily disciplines, while still contributing to broader comparative theories of embodiment, habitus, somatic attention, moral formation, and culturally mediated self-regulation.

The central implication is that psychological science should treat ritual, institution, and culture not merely as external contexts of already-formed minds, but as formative conditions of mind itself. Confucian self-cultivation demonstrates how a cultural tradition can organize bodily practice into a durable architecture of attention, affect, habit, and moral agency. This makes it valuable for theoretical and philosophical psychology, especially for debates over embodied selfhood, moral development, culturally mediated self-regulation, and psychological change.

## 7 Conclusion

This study has proposed the Confucian Embodied Self-Cultivation Model as a theoretical account of psychological transformation grounded in ritualized embodiment. Through close readings of Confucian classical formulations and a historically situated illustration from Huizhou lineage ritual, the article has argued that Confucian cultivation can be theorized through a layered mechanism: embodied moral agency, ritualized attention and affect regulation, repeated embodied self-cultivation, immanent psychological transformation, and cultural-institutional mediation.

The central contribution is to shift attention from the classificatory question of whether Confucianism should be defined as religion to the psychological question of how Confucian practice may contribute to psychological transformation. In the configuration examined here, ritual does not merely symbolize values; it trains bodies, disciplines attention, regulates affect, and stabilizes habits. Moral subjectivity becomes durable because it is repeatedly enacted in forms that are spatially organized, socially monitored, and institutionally sustained.

The model is analytically delimited. It does not claim that all Confucian traditions operate in the same way, nor that Huizhou represents Confucianism as a whole. Rather, Huizhou provides a high-resolution historical case in which the mechanism of embodied self-cultivation becomes especially visible. The model should therefore be treated as a comparative tool for examining when and how analogous configurations of ritualized embodiment, institutional repetition, and moral transformation appear across different cultural contexts.

For theoretical and philosophical psychology, the broader implication is that selfhood and moral agency are not formed only through private cognition, explicit belief, or abstract reasoning. They can also be shaped through disciplined bodies, ritualized practices, and institutional continuities. Confucian self-cultivation thus offers a non-Western model of psychological transformation, expanding comparative theories of embodied cognition, moral psychology, self-regulation, cultural psychology, and culturally mediated personhood.

## Data Availability

The original contributions presented in the study are included in the article/supplementary material, further inquiries can be directed to the corresponding author.
